# Improving mental health literacy among young people aged 11–15 years in Java, Indonesia: the co-development of a culturally-appropriate, user-centred resource (The IMPeTUs Intervention)

**DOI:** 10.1186/s13034-021-00410-5

**Published:** 2021-10-07

**Authors:** Helen Brooks, Armaji Kamaludi Syarif, Rebecca Pedley, Irman Irmansyah, Benny Prawira, Karina Lovell, Cicih Opitasari, Adam Ardisasmita, Ira Savitri Tanjung, Laoise Renwick, Soraya Salim, Penny Bee

**Affiliations:** 1 Division of Nursing, Midwifery and Social Work, School of Health Sciences, Manchester Academic Health Science Centre, University of Manchester, Manchester, UK; 2 National Institute of Health Research and Development, Ministry of Health, Jakarta, Republic of Indonesia; 3 Marzoeki Mahdi Hospital, Bogor, Republic of Indonesia; 4 Into the Light, Jakarta, Republic of Indonesia; 5 Arsa Kids, Jakarta, Republic of Indonesia; 6 Pulih@thePeak-Women, Youth and Family Empowerment Centre, Jakarta, Republic of Indonesia

**Keywords:** Indonesia, Mental health literacy, Children and young people, Intervention development, Co-production, Patient and public involvement

## Abstract

**Background:**

Many mental health problems emerge in late childhood and contribute significantly to the global burden of disease. Adverse outcomes can extend into adulthood if left untreated. This impact is exacerbated in low- and middle-income countries where significant treatment gaps persist. Improving mental health literacy may offer an effective strategy for early intervention but remains underexplored in these contexts.

**Methods:**

An intervention was co-developed with children and young people (CYP) by undertaking a needs analysis combined with stakeholder consensus activities. A systematic review of mental health literacy interventions in South-East Asia was undertaken in addition to semi-structured interviews with 43 children and young people (19 with and 24 without experience of anxiety and depression), 19 parents of children with experience of mental health problems and 25 education and health professionals. A focus group was also held with 8 key stakeholders immersed nationally in policy and practice. Interview schedules explored participants’ experiences of mental health, unmet needs and priorities for intervention. Data were synthesised and presented at a 3-day co-production workshop. Attendees included 13 CYP, 6 parents/guardians, 2 teachers, 8 health professionals, 2 academics and 3 game designers. Consensus exercises were utilised to identify the preferred format, content and delivery of the intervention. A smaller group of patient and public involvement contributors worked with designers to further iterate the intervention.

**Results:**

An immersive storyline digital application was co-developed for young people aged 11–15 with the primary aim of improving mental health literacy and self-management. The intervention comprises two chapters; one depression focussed, and the other anxiety focussed. The storyline format is interspersed with interactive games and exercises to promote engagement and encourage self-management. CYP also take part in group sessions delivered by trained facilitators before and after intervention use to discuss outcomes of and issues raised during the game.

**Conclusion:**

The IMPeTUs intervention has been co-designed for CYP aged 11–15 to improve mental health literacy and enhance self-management abilities. To the best of our knowledge, this is the first Indonesian digital intervention to improve mental health literacy and self-management for this population. Implementation, acceptability, and impact are currently being explored in a multi-site case study evaluation.

**Supplementary Information:**

The online version contains supplementary material available at 10.1186/s13034-021-00410-5.

## Background

Mental health problems, such as anxiety and depression, can impact negatively on daily functioning and physical health, increase mortality and challenge productivity and sustainable development in low- and middle-income countries (LMICs) [1]. They frequently co-occur with each other and with chronic physical health conditions which can substantially worsen health outcomes [2]. Mental health problems also account for a significant proportion of disease burden in children and young people (CYP) with an estimated 10–20% affected worldwide indicating that this group would benefit from increased public health attention [3].

Indonesia, a lower-middle income country in South-East Asia, has a population of over 270 million making it the fourth most populated country in the world. Mental health problems are highly prevalent and depression alone has been identified as the second highest cause of disability in Indonesia. Long-standing deficits in treatment access persist [4]. Disability-adjusted life-years^1^ due to mental health problems have increased in Indonesia over the last decade, with a 22% increase in depressive disorders and an 18% increase in anxiety disorders [5]. Amongst young people in Indonesia, mental health problems account for approximately one quarter of the disease burden and suicidality is a substantial and ongoing concern [4, 6]. There is also considerable evidence that depression and anxiety can increase after public health emergencies such as natural disasters or global pandemics [7]. This is exacerbated in lower resource settings which has left LMICs disproportionately vulnerable to the mental health effects of the COVID-19 crisis.

A central goal of the World Health Organisation’s sustainable development strategy is to promote mental health and wellbeing and ameliorate the burden of non-communicable diseases in order to reduce premature mortality by one third by 2030 [8]. Significant and persistent treatment gaps in LMICs have given rise to an urgent need to develop scalable and effective interventions to improve mental health and wellbeing. In Indonesia, mental health is now a national priority, but community mental health services remain embryonic. As in other LMICs, stigma towards those with mental health problems combined with erroneous beliefs about mental health problems further contribute to delays in access to effective care [9].

There is growing evidence demonstrating the importance of mental health literacy—‘the knowledge and beliefs about mental disorders which aid their recognition, management or prevention’ [10]—to a range of health and functional outcomes [11]. Mental health literacy comprises: the ability to recognise mental health problems, being able to understand the causes of mental health problems, knowledge about self-help and professional support and beliefs about the factors affecting help-seeking [10]. In older adults, low levels of mental health literacy have been found to be associated with higher mortality rates [12]. Systematic reviews examining the effectiveness of mental health literacy interventions for adolescents demonstrate their potential value as health promotion and prevention tools [13, 14].

Despite this, mental health literacy in CYP and LMIC populations is comparatively underexplored. Results from recent evaluation studies suggest that school and community based initiatives targeting specific mental health literacy components hold promise [13, 15]. A recent systematic review suggests that mental health literacy is low amongst CYP in LMICs and highlights the need for culturally appropriate interventions to support young people in this regard [16]. In Indonesia, particularly, low levels of mental health literacy are pervasive which is likely to contribute to the development of common mental health problems and impact negatively on timely access to treatment [17, 18]. Qualitative evidence in Indonesia which has identified stigmatising views of mental health problems amongst CYP and highlighted the salience of religion as key determinants of mental illness further demonstrates the need for culturally sensitive mental health literacy interventions to optimise acceptability and buy-in from young people [9].

In terms of the format of interventions, CYP prioritise support from lay networks including peers and family members over formal support networks such as health professionals [16, 19]. The foci of previous interventions have tended to be on school based initiatives in high income and western countries. Whilst these interventions have demonstrated effectiveness in terms of improving mental health literacy [20], they are unlikely to reach all CYP who could benefit from interventions due to a lack of cultural sensitivity and the limited delivery settings. They are also unlikely to adequately incorporate parents and the wider community which is likely to be important in the collectivist cultures associated with South-East Asia in which individuals are considered interdependent with family, community and society as a whole.

This paper reports on the development of the IMPeTUS intervention designed to improve mental health literacy amongst children and young people aged 11–15 in Java, Indonesia [21]. This intervention was co-developed with CYP, parents and professionals in Indonesia. Additionally, a systematic review undertaken as part of phase 1 identified a striking lack of culturally sensitive mental health literacy interventions relevant for use in Indonesia which necessitated the development of a new intervention. The development of our intervention followed the Medical Research Council Guidelines for the development and evaluation of complex interventions [22] and is closely aligned with World Health Organisation recommendations to enhance mental health resources in schools.

Initial Patient and Public Involvement (PPI) consultation undertaken during grant development activities suggested an interactive design which included some group-based components in addition to individual interaction with the intervention. Recent reviews of the literature indicate that interventions which incorporate interaction through the use of games, simulations and group work can be more efficacious than purely psychoeducation based initiatives [23]. The proposed structure of the intervention drew on data from a systematic review and qualitative interviews with multiple stakeholders in addition to current evidence which demonstrates the efficacy of short-term interventions for health promotion [24, 25].

## Materials and methods

The co-development of the intervention drew on principles of Experience Based Co-Design [26] and involved 3 phases [27]:

- Needs analysis—a systematic review of existing literature and primary qualitative data collection with multiple stakeholders to identify intervention format, content and implementation priorities.
- Stakeholder consensus—a 3-day co-production workshop with 50 attendees to agree the format and provisional content and delivery approaches.
- Intervention development and refinement—sustained group work between intervention designers and members of the research team and PPI advisory group.

The manuscript follows the guidance for reporting intervention development studies in health research [28]. Activities and findings from each phase of development are presented below.

### Patient and Public Involvement (PPI)

At study outset we convened a project advisory panel in Indonesia with support from project partners, KPSI, Into the Light and The Pulih Foundation. For more details on these individual groups see Brooks et al. [21]. The panel comprised 8 participants including 3 young people, 1 parent, and 4 health and education professionals and non-governmental representatives. All PPI contributors received a research methods training course alongside study researchers to facilitate involvement and engagement over the course of the study. The training course is the NICE shared learning database and was adapted for use in Indonesia [29].

Ethical approval for the study and all documented procedures was granted by University of Manchester Research Ethics Committee (Ref: 2018-4949-7908) and The Ministry of Health Indonesia (Ref: LB:02.01/2/KE.201/2019).

### Phase 1: Needs analysis

#### 1a Systematic review

We undertook a rapid evidence synthesis to identify interventions that had previously been used to target mental health literacy or depression and anxiety focussed self-management for CYP in South-East Asia. The synthesis aimed to ascertain their effectiveness in addition to any contextual or delivery characteristics which impacted on their acceptability, impact, and implementation. Full details of the review are available on PROSPERO [https://www.crd.york.ac.uk/prospero/display_record.php?RecordID=108883—PROSPERO 2018 CRD42018108883] and in Brooks et al. [21].

Relevant databases (PsycINFO, MEDLINE, Embase, Cochrane Central Register of Controlled Trials (CENTRAL), Scopus, Cumulative Index to Nursing and Allied Health Literature Plus (CINAHL Plus), Social Sciences full texts, ASSIA, ERIC, SCI and SSCI) were searched to identify published quantitative, qualitative, mixed methods studies and unpublished grey literature. We reviewed reference lists of included papers, specific author searches and forward-citation tracking of included papers. We also undertook a comprehensive grey literature search in collaboration with local partners which involved searching grey literature databases (such as OpenGrey) and google searches for relevant websites. Included papers were assessed for quality using Pluye et al.’s quality criteria which is suitable for critically appraising qualitative, quantitative and mixed methods papers [30].

Papers were eligible for inclusion if they were published in English or Bahasa Indonesian (the primary language in Indonesia), had a primary focus on mental health literacy or anxiety and depression focussed self-management interventions for CYP aged under 18 and were conducted in South-East Asia. Interventions could be undertaken in any setting. We did not include articles published in other languages.

Two reviewers independently screened title and abstracts and full texts of included papers with conflicts resolved by a third reviewer. The screening process was managed within Covidence (www.covidence.org). Data extraction was supported by Microsoft Excel to allow data relating to study context, participants, intervention, design/content and intervention outcomes to be collated. Data was double extracted by two researchers with independent extractions cross-checked for accuracy.

Given the heterogeneity of included studies in terms of intervention content, delivery and implementation, a meta-analysis was not possible. A narrative synthesis of included studies was therefore undertaken.

#### 1b Primary research: qualitative interviews and focus groups

Primary data collection was undertaken across three study sites in Java: Jakarta, Bogor and Magelang. These three settings were selected to represent differing cultures, levels of urbanisation and service infrastructure. All three sites also had some form of child and adolescent mental health service provision to facilitate recruitment.

A range of stakeholders, including children and young people, parents, health and education professionals and national key informants (those immersed at a national level in health or education policy and practice), were invited to take part in the primary research component of developing the mental health literacy intervention. Participants were recruited either via direct invitations from primary care services, third sector organisations, schools and child and adolescent health services (CYP and parents) or direct invitations from the study team (professionals/key informants). Participants who were interested in taking part in the study contacted the research team directly to discuss participation. Participants were purposively sampled in relation to age, gender and geographical location to ensure sample diversity and to incorporate a range of inputs [21].

Semi-structured interviews were undertaken with 43 children and young people aged 11–15 both with and without lived experience of anxiety and depression, 21 parents of children with experience of mental health problems and 25 professionals (education and health professionals). This age range was chosen at the advice of our Indonesian collaborators as it reflected the junior high school population in Indonesia who range from 11–15 years old. We also undertook a focus group with key stakeholders at a national level who were considered influential in the education or provision of care for CYP such as government ministers, policy makers, service directors or community leaders.

Written consent was obtained from all participants prior to data collection commencing. CYP could only participate once written assent was obtained in addition to written parental/guardian consent. Interviews were conducted between November 2019 and January 2020 lasting between 20 and 40 min. For more details on data collection procedures see [21]. Participants were asked to complete a brief demographic form prior to participation. Travel expenses were reimbursed and CYP and parental participants received compensation for their time.

Interview schedules were developed in consultation with the PPI advisory group and all data was collected by Indonesian researchers [21]. Interviews/focus groups schedules and included terminology were adapted for each stakeholder group but covered similar topics such as previous support from mental health services, unmet needs, factors that may potentially impact on the implementation of the intervention, treatment gaps and priorities for intervention content, design and implementation. Interviews/focus groups were digitally recorded and transcribed verbatim before being translated into English to support analysis. Five per cent of translations were checked to ensure accuracy [31].

Data were analysed using framework analysis to identify priorities for the content, design and implementation of the intervention using components of the TiDIER checklist for intervention description [32].

### Phase 2&3: Stakeholder consensus and intervention development and refinement

Stakeholder consensus was undertaken drawing on principles of the Experience-based Co-design Toolkit [26]. As part of the process of co-developing the intervention, a 3-day synthesis and co-production workshop was held in Bekasi, Indonesia in December 2019. The aim of this workshop was to review findings from the literature review and primary data collection and to agree on the details of a provisional design brief and implementation strategy for the intervention. A total of 50 participants attended the workshops. These included the research team and PPI advisory group (n = 14), 13 CYP aged between 11–15, 6 parents/guardians, 2 teachers, 8 mental health professionals representing all three study sites, 2 teachers, 2 academics and 3 game, web and graphic designers.

During the 3-day event we presented findings from both the systematic review and primary data collection and used consensus exercises (e.g. vote counting) to confirm prioritised format identified during phase 1 and to reach consensus on the details of the specific components of the intervention. Where discrepancies arose between stakeholders’ views and collected data, these were explored and discussed and voting exercises were repeated. An overview of workshop activities can be found below with further detail provided in Appendix 1:

Initial co-design event: This research team and the PPI advisory group attended all 3 days of the event.

- Day 1: Attendees in addition to the research team and the PPI advisory group included mental health professionals representing the three hospitals involved in the research and 13 CYP (some of whom attended with their parents or guardians) and had not been involved in the primary research undertaken as part of the needs analysis; game designers and graphic artists also attended. Initial activities included an icebreaker exercise and getting to know you session for the CYP, an introduction to the wider study and basic information about mental health using vignettes developed by mental health professionals in order to orientate CYP to the topic area as recommended by project partners. The CYP then participated in facilitated creative activities and discussions to ascertain their preferences for intervention content, format, delivery, promotion and implementation. This included the use of arts activities such as drawing, model making and sculpting with playdough. In order to obtain consensus amongst CYP, children voted using smiley faces on flip charts to identify their preferred options. CYP participants attended day 1 only.
- Day 2: Attendees in addition to the research team and the PPI advisory group included health and education professionals and representatives from the Ministry of Education. Key findings from the needs analysis were presented to all stakeholders including the priorities identified by CYP on day 1 in order to promote discussion about the proposed intervention. Next, participants were split into mixed groups to discuss their preferred intervention content, format, delivery and implementation options. One member of each individual group presented an overview of their discussion to the wider group. All stakeholders were then given the option to vote on their preferred intervention options following these group presentations.
- Day 3: Attendees in addition to the research team and the PPI advisory group included game developers, web designers and graphic artists. The results from day 1 and 2 were presented and discussions were centred on how best to design the proposed intervention and culminated in an agreed design brief for the game designers to develop.

After the initial co-design event, the game designers continued to work closely with the research team and PPI advisory group on an ongoing basis to iterate and finalise the intervention. There were three further face-to-face meetings. The first meeting finalised the intervention design including the plans for intervention content such as the exercises and games to be included. The second meeting involved presenting the prototype intervention to the research team and PPI advisory group for further input. The third meeting involved presenting the revised prototype and allowed testing by all members of the research team and the PPI advisory group and further feedback was provided. The final intervention ready for case study evaluation was presented to the research team and PPI advisory group during a celebration event which was held online following the outbreak of Covid-19.

## Results

### Phase 1

#### 1a Systematic review

A total of 7715 title and abstract records were assessed against the eligibility criteria, of which 128 records progressed to full text screening. After full text review, 6 publications across 5 studies were eligible for inclusion [33–38]. Studies included 4 quantitative and 1 mixed methods study, conducted across 3 South-East Asian countries (Singapore n = 2, Thailand n = 2, Cambodia n = 1).

Quality scores range from 0%–58.3% (See Additional file 1), with the single mixed methods study achieving the highest score [33]. Of the four quantitative experimental studies, two of the four studies (reported across five manuscripts) scored 0% on all indices, indicating poor quality [36–38]. The remaining two quantitative studies scored 33.3%; both failing to conduct (or report) appropriate sequence generation and/or randomization or allocation concealment and/or blinding [34, 35].

Of the 5 studies, only 1 focused on mental health literacy [37]; the remainder were self-management interventions. Interventions were delivered in schools across all studies. Interventions were aimed at reducing anxiety [33], preventing test anxiety [35], improving coping skills and mental health [36, 38], reducing risk factors for suicide [34] and improving coping with stress [37]. Only a single study, conducted in Singapore, reported on intervention acceptability [33].

Four studies reported improvements to CYP’s self-management, knowledge or mental health literacy as a result of receiving the intervention [33, 34, 36–38]. CYP who had received the intervention from intensively trained teachers (but not those whose teacher received lower intensity training) improved in terms of their coping skills at 1-month follow up, compared to the control group [36, 38]. A study conducted in Cambodia found that all CYP reported some benefits, but that girls showed mild/moderate improvement across 3/4 life skills dimensions (interpersonal communication/human relationship, physical fitness/health maintenance and total life skills) whereas boys only showed improvement in the interpersonal communication/human relationship dimension [34]. The pattern reversed when boys and girls who were at ‘high risk’ of suicide were analysed separately, with boys improving in skills across several domains and girls showing no benefit in any area [34]. One study reported improvements to CYP’s ‘knowledge attitudes and practice’ but little detail was reported as to what the measure was designed to capture [37]. The single study using mixed methods found through semi-structured interviews that 79.3% of parents thought their child had independently applied coping strategies they had developed through the intervention (e.g. deep breathing) and that 45.5% had improved in terms of their wellbeing or ability to manage their fears [33].

All studies showed that CYP showed some improvements in terms of mental health symptoms after receiving the intervention [33, 35–38]. Benefits in one study were however restricted to particular subgroups of young people; when all youth were analysed, effect sizes on the Youth-Self report were small/no effect, whereas a small effect of the intervention across several domains was found amongst boys at high risk of suicide (but not girls) [34].

The single study which evaluated intervention acceptability found that staff were highly satisfied with training and that most parents thought the intervention was useful (87.9%) and enjoyable (81.8%) [33]. Based on a staff review session, a number of factors which facilitated implementation were identified, for example strong and consistent support from stakeholders (66.3% of comments) and appropriate/meaningful resources (7.14%). Barriers to implementation were also identified, for example, 20.37% of staff mentioned scheduling difficulties as a barrier and 16.6% mentioned lack of parental support, due to parents being unwilling to reveal their child’s fear. CYP qualitative views on acceptability were not included in the study.

#### 1b Primary data collection: qualitative interviews with multiple stakeholders

A description of findings relating to framework components can be found below. Supporting quotes are provided in Appendix 2.

### Perceived value of the intervention

The vast majority of CYP, parents and professionals thought it would be helpful to develop an intervention to promote mental health literacy and encourage self-management. Participants who took part in the study felt strongly that CYP in Indonesia would access such an intervention and that it could be a useful tool for CYP, parents and teachers alike.

Stakeholders coalesced in their reasons for attributing value to such an intervention which included: the opportunity to increase awareness and knowledge about mental health and promote reflection, provide hope in relation to recovery, enhance individual resilience, and support the development of appropriate self-management strategies which could ultimately impact on mental health. In order to have a positive impact, stakeholders felt that the game needed to be appropriately designed to be understandable to CYP and suitably engaging.

One parent and two CYP were not convinced of the value of such interventions. Reasons for this include the potential distraction from schoolwork, that interventions of this type would not be of interest to CYP and the potential for CYP to become addicted to interventions if they were delivered using technology.

### What should the intervention include?

CYP priorities for intervention content centred on the provision of information about mental health in order to promote wellbeing. Parent and professional stakeholders included additional suggestions for content including focusing on issues that currently affected CYP in Indonesia such as religion/morality, bullying and divorce. They also considered information about mental health to be important, as well as to self-management strategies, content about school and how to socialize with others, issues relating to family life, and how family members can support CYP.

The majority of CYP thought the format should contain animations or cartoons primarily because these were considered to be the most engaging for CYP and easily understandable. Parents and professionals agreed gave additional suggestions such as movies, testimonials and quizzes.

### What format should the intervention take?

Stakeholders coalesced in their shared preference for a game format and agreed this should be delivered digitally. They also highlighted that the intervention should not be purely didactic nor be too long in duration. Reasons for this preference were shared amongst stakeholders and included the interactive and fun nature of games which would promote engagement amongst young people whilst at the same time being educational.

### Who should provide the intervention in which settings?

All stakeholders felt that the game would be delivered optimally in school settings, but other options included formal health services, home settings and community venues. Parents and professionals thought that the intervention could also be provided by parents or health professionals, but this was not raised by children themselves.

### Mode of delivery?

All stakeholders thought that the intervention should be delivered using a form of current technology which would allow children to use it individually or in a group, e.g., via a mobile phone. This was because stakeholders felt this mode of delivery was most aligned to CYP current practices and preferences.

### Phase 2&3: Stakeholder consensus and intervention development and refinement

#### Prototype outline

In line with the TIDieR Checklist [32], the qualitative data provided consensus on the high-level features of the IMPeTUs mental health intervention (e.g. format, delivery setting etc.). These were confirmed with attendees at the stakeholder consensus workshops as well as reaching consensus on the specific details of each of the higher-level features identified during the primary research component of the intervention development process (See Table 1).

**Table 1:** Detail on the proposed IMPeTUS intervention

Agreement	Inconsistencies/further evidence required
Aim: To increase mental health literacy and develop self-management strategies Content: Interactive digital story-line game combined with a facilitated group discussion before and after use of the intervention. Feedback to be provided by the intervention to CYP in terms of their responses Target users: CYP aged 11–15 Where: Setting agnostic. Should be able to be used in schools/mental health services and third sector organisations as a minimum Delivery: Individual game play and game play with parents. Facilitated group sessions. Facilitator dependent on delivery setting Outcomes: Individual skills and knowledge (MHL), mental health (anxiety and depression), family cohesion, quality of life Resources: Digital game, links to further information about specific issues raised in the game (web based), facilitator training, implementation guidance, resource for parents Training: Group facilitator training, training for implementers	• Need for group discussions before and after intervention engagement to discuss issues raised in the game—CYP preference • Optimal implementation context (school/community organisation/third sector organization—evaluation required as no consensus

**Fig. 1:**
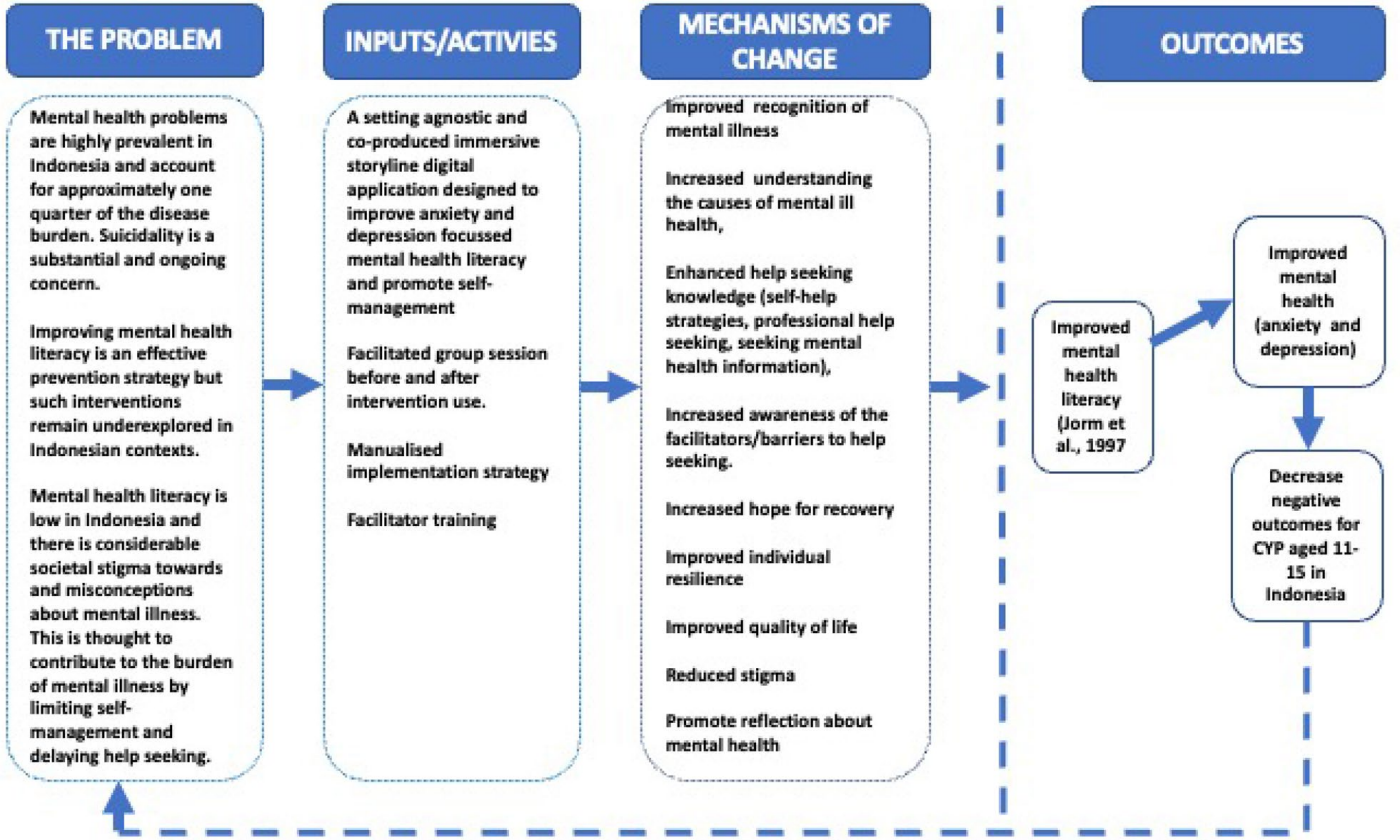
Draft theory of change for the impetus intervention

The prototype intervention is an immersive storyline digital application co-developed with young people aged 11–15 to improve mental health literacy and develop self-management strategies (Appendix 3). The intervention comprises two chapters; one which targets depression and the other which targets anxiety. The storyline format is interspersed with interactive games and exercises to promote engagement and encourage self-management. The decisions young people make during the game impact the outcomes of the game. Stakeholders, and particularly CYP, felt it was important to supplement the digital game with facilitated group discussions to provide a safe space to talk about the issues raised in the game and ask questions that may arise. Associated training materials for facilitators were also co-developed. For a draft Theory of Change underpinning the intervention, see Fig. 1. The prototype has been fully developed and is now ready for pilot testing and evaluation.

The primary aim of the intervention is to improve mental health literacy and anxiety and depression focussed self-management. The target population is CYP aged between 11 and 15 years of age. Minimum levels of intervention require engagement in both chapters of the intervention for a minimum of 1 h each and participation in a facilitated group session before and after the intervention (minimum 4 h in total).

## Discussion

To the best of our knowledge, this is the first study to co-develop a mental health literacy and self-management intervention for CYP in Indonesia. Indonesia is a lower-middle income country with high treatment access inequalities and mental health burden which demonstrates the need for low-cost scalable interventions to promote positive mental health [39].

Mental health problems contribute to approximately 25% of the disease burden among young Indonesians and suicidal behaviour is a significant concern [4, 6]. Evidence suggests that increasing mental health literacy is a useful way to prevent mental health problems and improve mental health and wellbeing [13]. Mental health literacy interventions are closely aligned to global mental health initiatives such as the WHO MHGap programme which advocate for brief, low cost and scalable interventions in low resource settings and the WHO sustainable development goals which prioritise upstream mental health prevention and self-management approaches [8, 40].

Mental health literacy may have particular relevance in Indonesia given recent evidence which suggests poor mental health literacy is pervasive amongst CYP [17] but such interventions remain underexplored in Indonesian contexts. Further, the systematic review we undertook as part of the needs assessment demonstrated that high quality, robust evaluations of mental health literacy interventions in Indonesia are lacking. A burgeoning evidence base points to the acceptability of, and preference for, alternative mental health help-seeking strategies such as peer or family support among CYP globally [16, 19] and the potential efficacy of mental health literacy interventions in both high and low-income settings [20]. Stakeholders in the current study were almost unanimous in their consideration of the value of such interventions in Indonesian contexts and the likelihood that they would be acceptable to CYP. Such interventions were considered a good opportunity to improve knowledge about mental health problems and mental health in general, engender hope for recovery, improve resilience and support the development of self-help strategies which could all ultimately impact on mental health. However, there was clear agreement that such interventions would need to be carefully designed to ensure cultural and developmental acceptability.

There were similarities too in the preferences for the design, format and delivery of the intervention amongst different stakeholder groups which facilitated the development of the intervention. However, other stakeholders did not anticipate the strength of desire amongst CYP for opportunities to create safe spaces to discuss mental health and ask any questions they may have which they did not feel were available elsewhere in their lives. This may reflect the wider stigma towards people with mental health problems which is highly prevalent in Indonesia [9] and could be addressed through the targeting of both younger and older generations in future community engagement to drive attitudinal change and lessen stigma. Future implementation of the IMPeTUs intervention would also benefit from the development of close community and intersectoral partnerships in addition to the recruitment of local opinion leaders to draw on their influences to increase community awareness of mental health and maximise involvement in ongoing research activities [41].

The 2014 WHO Atlas survey shows that in Indonesia PPI infrastructure and activities have not kept pace with health systems development [42]. Despite a national appetite to implement effective PPI, current initiatives remain ad hoc. An explicit objective of our study was to co-produce our intervention with CYP and parents, health and education professionals and national key informants in close collaboration with our PPI advisory group who received training in research methods. PPI in our intervention development was facilitated by the close relationships and in-depth involvement from our three study partners: KPSI, Into the Light and The Pulih Foundation in addition to formal mental health services in each study site (see [21] for further information on study partners). Despite this, CYP from more rural and deprived areas were under-represented in our processes which may mean results are not transferable to these populations. Future studies should endeavour to promote societal understanding of PPI activities in Indonesia through public awareness campaigns and training for health researchers, clinical leads and non-governmental organisations to enhance public inclusion and diversity in mental health research and intervention design.

Recent evidence suggests PPI in health research was vastly diminished during the global Covid-19 pandemic [43]. Our project itself was paused for 12 months during the early stages of the pandemic and resumed whilst restrictions were still in place at study sites. This necessitated the shift in intervention development meetings from face-to-face to online platforms in order to deliver the project in accordance with agreed timelines. It also meant that some PPI members were older than the target age group when the study restarted, so four new members were recruited for the later meetings to supplement existing contributions. Maturity effects should be considered when CYP are involved in long-term projects particularly if there are disruptions to study progress.

However, the use of remote communication methods actually appeared to facilitate attendance at meetings and enhance meeting efficiency without placing any additional burden on study participants. This suggests that with shared commitment to the importance of PPI in research amongst project stakeholders and adequate communication strategies designed collaboratively with PPI partners, meaningful involvement and engagement can be sustained in spite of national emergencies. Hybrid models which incorporate some remote and some in person consultation could also be considered in future intervention development procedures especially in countries which cover large geographical areas such as Indonesia.

Our study has a number of limitations including the paucity of high-quality research identified in our systematic review. Participants who took part in the primary research components whilst sampled purposively were limited to three geographical areas of Java, Indonesia and findings may not translate to other areas of Indonesia. The self-selection of participants may also reflect some inherent bias in included data. Future work will necessitate the formal evaluation of the intervention including exploring its feasibility and acceptability to a wide range of children and young people prior to definitive evaluation of its clinical and cost effectiveness in a randomised controlled trial.

## Conclusions

There is a need for culturally appropriate and child-centred resources in Indonesia to promote mental health literacy and support self-management. We have utilised a co-productive approach which included working closely with children and young people and other stakeholders to optimise the development of the intervention and promote future engagement and utilisation. We will draw on these relationships in the future evaluation of the intervention using an in-depth mixed method case study approach and ensure future implementation remains appropriately child centred.

### Supplementary Information

**Additional file 1: Table S1.** Study characteristics. **Table S2.** Participants. **Table S3.** Intervention characteristics. **Table S4.** Outcomes. **Table S5.** Quality.

## Data Availability

The datasets generated and/or analysed during the current study are not publicly available due to ethical restrictions but are available from the corresponding author on reasonable request.

## Appendices

### Appendix 1 Initial co-design workshop schedules

#### 17th December 2019

Attendees:

- Research team
- PPI advisory group
- Designers
- Children and young people

**Table:**

Time	Activity	Lead	Details
10 a.m.	Welcome	Dr Irmansyah	Welcome to participants
10.15	Ice Breaker		Get participants to partner up and draw pictures of their partners with their eyes closed. CYP to each introduce their partner and show the group their picture
10.45	Presentation	Indonesian research team	Overview of PPI and its role in research. A discussion of the potential benefits for CYP and to research
11.00	Small group activity 1	Indonesian research team	Groups provided with prompt cards describing examples of research activities. In the set of cards, groups need to identify examples of • Children and young people participating in research (e.g. interviews/survey completion) • CYP being involved in the research process (co-production workshops/undertaking interviews etc.) • Examples of tokenistic involvement When decisions have been made cards are placed in the appropriate box on tables and then discussed with facilitator
11.45	Presentation	Indonesian research team	Overview of the study, PPI in the research process and of the planned intervention from needs assessment results
12 p.m.	LUNCH		
1 p.m.	Small group activity 2—how can an intervention improve mental health literacy?	Indonesian research team	Groups given a vignette of CYP living with anxiety/depression. Ask the group to identify issues within the vignette and for ideas about how a game could help them deal with the identified issues. Prompt cards to be provided for different mental health literacy components
1.30 p.m.	FEEDBACK	ALL	GROUPS TO FEEDBACK TO WIDER AUDIENCE
1.45 p.m.	Small group activity 3—format of the game	Indonesian research team	Groups given prompt cards with pictures of different types of games (card game, board game, digital application etc.) Groups to agree their preferred option for game format and reasons for this and then feedback to the rest of the group Voting exercise to identify priorities (four emoji faces places around the room (sad, neutral, happy and really happy) Read out ideas and CYP go to the emoji which best reflects their feeling on the idea Count the number at the really happy face emoji and the take the top scoring option forward
2.30 p.m.	Small group activity 4—What should the game look like?	Indonesian research team	CYP to draw pictures, use play dough, or use other crafts, write suggestions or use photos or online images to make suggestions to the wider group about what the game should look like
3.30 p.m.	Small group activity 5—What else would you need to accompany the game on a website?	Indonesian research team	Group to come up with ideas and put post-it notes on flip chart paper If time, get group to agree on priorities by voting (show of hands)
4 p.m.	Evaluation and next steps	Indonesian research team	The groups to be told what the next steps for impetus are Evaluation: Three flip chart pages titled what did you like, what did you not like, how could events be improved going forward are placed around the room. CYP to write what they liked, did not like or suggestions for improvements on post-it notes and place on the appropriate flip chart sheet
4.30 p.m.	CLOSE		

#### 18th December 2019

Attendees:

- Research team
- PPI advisory group
- Designers
- Teachers, clinicians, parents and other key stakeholders

**Table:**

Time	Activity	Lead	Details
9 a.m.	Welcome	Dr Irmansyah	Welcome to participants
9.15 a.m.	Presentation	Dr Irmansyah	Introduction to IMPeTUs and concept of mental health literacy
9.45 a.m.	Presentation	Indonesian Research team	Presentations of key findings of two systematic reviews
10.15 a.m.	Discussion	All	Group feedback/opportunities for questions
10.45	Break		
11 a.m.	Presentation	Indonesian research team	Presentation and discussion of key findings from qualitative interviews with each stakeholder group (CYP/parents/professionals/key informants)
11.45 a.m.	Discussion	All	Group feedback/opportunities for questions
12.15 p.m.	Presentation	Indonesian research team	Presentation and discussion of format/features/design of game and digital resources as agreed by children and young people during day 1
12.15 a.m.	Discussion	All	Group feedback/opportunities for questions
12.30 p.m.	Lunch		
1.30 p.m.	Small group work	Indonesian research team	Additional suggestions around format/features/design of game
1.45 p.m.	Feedback	All	Groups feedback to wider participants and consensus activities
2 p.m.	Small group work	Indonesian research team	Suggestions for additional resources that parents/teachers/clinicians would need to facilitate the game
2.45 p.m.	Feedback	All	Groups feedback to wider participants and consensus
3 p.m.	Small group	Indonesian research team	Suggestions for optimal implementation
3.45 p.m.	Feedback	All	Groups feedback to wider participants – consensus activities
4 p.m.	Next steps	Dr Irmansyah	Next steps for IMPeTUs outlined
4.15	Evaluation	All	Three flip chart pages titled what did you like, what did you not like, what could have been improved. Participants to write suggestions on post-it notes and place on relevant flip chart papers
4.45	Close		

#### 19th December 2019

Attendees:

- Research team
- PPI advisory group
- Designers

Group meeting: 9 a.m.–12 p.m. To discuss workshop results and agree final design brief for the IMPeTUs intervention.

### Appendix 2 Supporting quotes

**Table:**

Framework component	Supporting quotes
Perceived value of the intervention	*There needs to be some promotion of mental health from, from somewhere. [] A tool like that… Yes, it would help, it would help a lot.* **B316, Parent, Bogor** *Do you think this kind of tool would be helpful for young people?* *Yes… We can learn about mental health.* **C210, CYP, Magelang** *The intervention, it could help, it [could] help to maintain someone's mental health and then maybe—And then if you make a DVD, people will know different types of mental illness.* **B105, CYP, Bogor** *Like a short movie about someone who got better after having mental health problems. So that they can see—they get information that they see with their own eyes—someone who changes after they get better.* **B320, Parent, Bogor** *I think [an intervention would be] helpful because it would make them think.* **A420, Professional, Jakarta** *They’d just want to study less and less. I think games make children stupid, they make children not want to pray or study.'* **C323, Parent, Magelang**
What should the intervention include?	*Like, illustrations so that you don't get depressed, so that they don't have mental health problems* **C108, CYP, Jakarta** *Include content about school and how to socialise with others.* **A317, Parent, Jakarta** *Information regarding the criteria of people with mental health disorders, but not just putting the definition [in text], but showing it in the animation. Maybe there can be a teenager that does this and that, and then they become sad, or become something else. And then the teenager can maybe go to talk to a young counsellor or a counselling teacher, and they can say, my mental health problem is like this and that.* **C427, Professional, Magelang** *It should have nice colours. The video should be funny, and there needs to be laughs. Use cartoons.* **C213, CYP, Jakarta** *So there needs to be a real illustration of the reality, like maybe something like farewells, graduations. Talking about mental health and then there should be some illustrations that show good kinds of friends, and bad ones. There needs to be a scene of a parent telling their kid.* **A425, Professional, Jakarta**
What format should the intervention take?	*Audio… and visual. Like, games… gadgets.* **B103, CYP, Bogor** *I think a game would be great, a game and a DVD so that she doesn't play games all the time. She can watch the video some time, something like Upin & Ipin. And social activities and things like that.* **B319, Parent, Bogor**
Who should provide the intervention in which settings?	*Schools. Hospitals as well, but schools are better.* **C321, Parent, Magelang** *I think you have to promote it to schools first, because schools are a great filter to determine whether [the helping tool] is suitable for their students. If you go promote it to schools, then the parents will also know about it.* **C322, Parent, Magelang** *That’s a parent’s role. It’s a big role, and it’s important for parents to have someone or something they can refer to.* **C425, Professional, Magelang**
Mode of delivery	*Because people my age do everything on their gadgets, so getting information *via* a DVD or a game would be more attractive than reading books.* **B107, CYP, Bogor** *Kids these days always play on their phones or their computers.* **A314, Parent, Jakarta**

### Appendix 3 Screen shots and design information from the IMPeTUs intervention

Based on a needs assessment and primary research relating to mental health literacy in Indonesia, the Arsa Kids team co-designed with children and young people an immersive story line digital application with the purpose of increasing mental health literacy and developing anxiety and depression focused self-management strategies. The game was designed by our CYP clinical expert and pedagogical researcher at Arsa Kids, Putri Puspitaningrum.

This feature is designed to make the game more immersive by letting players personalize their character. They can input their name, personalise facial characteristics and clothing and enter what level of education they have.

The main feature of this game is the story. Players will have to make decisions based on particular situations. This situation will simulate real-life situations, whether someone already has a level of understanding about mental health or not. Each decision gives players different consequences, and they will have a different endings based on the outcomes of the different scenarios.

We also designed a number of mini-games to help players learn about positive mental health and illness in a more in-depth manner.

With this mini-game, the player will have to filter which information is accurate and which is not. They will answer questions about what to do when facing certain emotions or symptoms of mental illness. It will help them understand what to do when they encounter a problem and help them identify an appropriate response.

This mini-game is a simulation of a video game. This mini-game illustrates how distraction techniques can be used to challenge negative emotions.

This mini game is journaling. It provides another example of a useful self-management strategy.

Talking to counsellors or seeking professional help is uncommon in Indonesia. This game will help to normalise consultation with professionals to promote future help-seeking. Diversity of included characters is an in-built feature of the intervention.

In this mini-game, we teach CYP one of the techniques that can be used to promote calm when facing anxiety. This technique is called grounding, where you observe objects around you and focusing on a specific things, such as “finding an object with the colour of blue.”

This mini-game teaches another technique which is called the 4–7-8 breathing technique. It will help students when facing challenging emotions or difficult experiences.

Another technique introduced in this game is called “safety network habit.” This game will ask the player to identify influential people in their life who can protect them (like family, close friends, teachers, neighbours, health professionals, other community members etc.). They will put the name of their chosen people on each finger.

The last game is a puzzle game to visualize emotions such as happiness, sadness and fear. It will help them to understand how to identify different kinds of emotions and how to help themselves and others.

## Footnotes

1 One disability-adjusted life-year or DALY corresponds to the loss of the equivalent of 1 year of full health.
