# Supplementary material for: Improving mental health literacy among young people aged 11–15 years in Java, Indonesia: the co-development of a culturally-appropriate, user-centred resource (The IMPeTUs Intervention)
Source: Child Adolesc Psychiatry Ment Health. 2021 Oct 7;15:56. doi: 10.1186/s13034-021-00410-5 (PMC8496433; doi:10.1186/s13034-021-00410-5)
Supplement: Supplementary file 1 — Additional file 1: Table S1. Study characteristics. Table S2. Participants. Table S3. Intervention characteristics. Table S4. Outcomes. Table S5. Quality. [file 13034_2021_410_MOESM1_ESM.docx]

**Additional file 1: Systematic review tables**

**Table S1: Study characteristics**

| **STUDY ID** | **COUNTRY** | **METHODOLOGY** | **DESIGN** E.G. RCT | **MEASURES OF MH LITERACY /KNOWLEDGE** |
| --- | --- | --- | --- | --- |
| Drmic 2017 | Singapore | Mixed | Single arm pre-post intervention study | None |
| Yeo, 2016 | Singapore | Quantitative | Two arm pre-post intervention study (non randomised/quasi-experimental) | None |
| Phuphaibul 2003 | Thailand | Quantitative | three arm pre-post intervention study ( children within intervention group schools were randomly allocated classes to intervention) | None |
| Phuphaibul 2005 | Thailand | Quantitative | three arm pre-post intervention study ( children within intervention group schools were randomly allocated classes to intervention) | None |
| Jegannathan 2014 | Cambodia | Quantitative | Two arm pre-post intervention study | None |
| Monkong 2009 | Thailand | Quantitative | Two arm pre post (quasi-experimental) | Knowledge, Attitude and Practice (KAP) of coping with stress by problem solving and emotional management** |

**Table S2: Participants**

| **STUDY ID** | **N TOTAL SAMPLE** | **AGE RANGE** | **M AGE (SD)** | **N (%) FEMALES** | **SAMPLE DESCRIPTION (E.G. YP WITH DEPRESSION, YP WITHOUT DIAGNOSED MH PROBLEMS ETC)** |
| --- | --- | --- | --- | --- | --- |
| Drmic 2017 | 44 | 13-15 | NS | 6 (14%) | CYP (drawn from across 22 mainstream secondary schools) with ASD and anxiety (meeting cut-off of at least one subscale score of the Screen for Child Related Anxiety Disorders) on total anxiety score or a sub-scale i.e. panic disorder, generalised anxiety disorder, separation anxiety disorder, social anxiety disorder or significant school avoidance) - child or parent version. |
| Yeo, 2016 | 115 (57 control; 58 CBT group) | 9-12 | 10.15 (0.50) | 45 (39%) | Young people drawn from 4 intact school classes (2 intervention classes; 2 control classes) |
| Phuphaibul 2003 | 1580 | NS - grades 7-9 | 13.72 (NS) | NS | School children from private and government schools grades 7-9. Experimental group 1 was drawn from 6 schools; experimental group 2 was drawn from 7 schools, control group drawn from 3 schools. Schools chose 100 students for pre and post test at random. |
| Phuphaibul 2005 | 1580 | NS - grades 7-9 | 13.72 (NS) | NS | School children from private and government schools grades 7-9. Experimental group 1 was drawn from 6 schools; experimental group 2 was drawn from 7 schools, control group drawn from 3 schools. Schools chose 100 students for pre and post test at random. |
| Jegannathan 2014 | Baseline N = 321, Follow up N = 299 (experimental = 168, control = 131) | NS - secondary school age | NS | 162 (50.5%)) | Young people drawn from whole school classes in secondary schools |
| Monkong 2009 | 84 (experimental =44, control = 40) | 15-18 | NS | 50 (59.5%) | Students drawn from two randomly chosen secondary schools in a Province of Thailand; one experimental school and one control |

**Table S3: Intervention characteristics**

| **STUDY ID** | **INTERVENTION TARGET/AIM E.G. ENHANCE MH LITERACY** | **Intervention setting/context** | **INTERVENTION CONTENT** | **CONTROL CONTENT (IF APPLICABLE)** | **NUMBER OF INTERVENTION SESSIONS** | **LENGTH OF INTERVENTION SESSIONS** |
| --- | --- | --- | --- | --- | --- | --- |
| Drmic 2017 | Reduce anxiety symptoms in youth with high functioning ASD. | Mainstream secondary schools, small groups (n=2-3 students) | Based on Facing Your Fears CBT protocol; delivered by non-clinicians (learning and behavioural support staff) who were coached by psychologists and trained across a 4-day course. Includes core CBT for anxiety in youth - psychoeducation, developing of coping skills (e.g. deep breathing), emotion regulation, problem solving, cognitive self-control (e.g. automatic thoughts), graded exposure. Delivered in small groups (2-3 students). + parents sessions involving understanding 'facing your fears' programme (its core components, exposure hierarchies, parental anxiety etc) and child follow up. | N/A - no control | YP = 10; Parents = 3 | YP = 1-1.5hr, Parents = NS |
| Yeo, 2016 | To prevent test anxiety through equipping young people with coping skills to manage anxiety ahead of exams | Four classes from public elementary schools, delivered during 'Form Teacher Guidance Period' during the month leading up to exams | Psychologist-delivered behavioural strategy focused preventative intervention with cognitive modification based on CBT principles including: psycho-education, relaxation training, self-instruction, exposure, skills training. Homework between sessions; audio CD/relaxation script. | Usual schooling | 4 (spread over 4 weeks) | 30 minutes |
| Phuphaibul 2003 | Improve coping skills and mental health of school children | private and government secondary schools | Module based on participatory learning principles and delivered by school teachers/ school nurses/counsellor and consisting of 1) instructions for users, 2) A video cassette depicting stories about a teen with life problems - students asked to critically analyse and discuss ways of coping; 3) guidelines for teachers, nurses, counsellors, 4) reference materials for students and users to facilitate discussions**. Experimental group I:** Teachers received intensive 2 day classroom based tuition in using module. **Experimental group II**: Teachers received less intensive 1/2 day training in the module. | Usual schooling - teachers were not trained/module not provided | Two sessions | 45 minutes each |
| Phuphaibul 2005 | Improve coping skills and mental health of school children | private and government secondary schools | Module based on participatory learning principles and delivered by school teachers/ school nurses/counsellor and consisting of 1)instructions for users, 2) A video cassette depicting stories about a teen with life problems - students asked to critically analyse and discuss ways of coping; 3) guidelines for teachers, nurses, counsellors, 4) reference materials for students and users to facilitate discussions**. Experimental group I:** Teachers received intensive 2 day classroom based tuition in using module. **Experimental group II**: Teachers received less intensive 1/2 day training in the module. | Usual schooling - teachers were not trained/module not provided | Two sessions | 45 minutes each |
| Jegannathan 2014 | Reduce risk factors for suicide among YP | 2 government secondary schools (1 experimental, one control) in Cambodia (6 classes randomly chosen from each school) | Enabling YP to make health choices, adopt health behaviour and reduce high risk behaviour through increasing 'life skills'. Life skills improved via 5 core skills -creative and critical thinking, decision making and problem solving, effective communication and interpersonal relationship skills, self-awareness and empathy and coping with emotions stress. Life skill modules related to suicide were chosen: motivation, concentration and improving memory, problem solving skills, peer pressure and saying 'no' to drugs/tobacco, coping with stress, facing changes/problems, self-esteem, sensation-seeking behaviour, self-awareness, understanding depression/suicide | 3 sessions on health, hygiene and nutrition e.g. hand washing, micronutrient deficiency | Six sessions delivered over 20-24 weeks with 1 or 2 sessions per week. | 90-100 mins |
| Monkong 2009 | To help adolescents cope with stress | 2 secondary schools | Based on cognitive-experiential self-theory and life skills technique. 'Training' in concept of thinking, problem solving strategies and controlling emotions. | Unclear | 5 days + 'booster program' every 15 days for 9 months | 5 days (booster session length not reported) |

**Table S4: Outcomes**

| **STUDY ID (Author last name, year)** |  |
| --- | --- |
| Drmic | 89% (n=39) of students completed the program, 5 students withdrew (due to absenteeism from school, student/parent opt out, student resistance to attend sessions). Follow up obtained for 35 students.  **PRIMARY OUTCOMES (outcome in bold appears to pertain to primary outcome in terms of self-management)**: Parent impressions semi-structured interview: The majority of parents thought the intervention was useful (87.9%) and enjoyable (81.8%) and that strategies could be carried out independently (78.8%). 93.3% of parents thought there was some observable reduction in anxiety and "a**n increased ability to manage their fears and/or an improvement in wellbeing and behaviours** (45.5%). Parents thought that child worries and behaviours had improved. **Most (79.3%) reported that "their children independently applied some of the coping strategies learned in the program, such as deep breathing, and using the colour coding to verbalise his stress/anxiety level**"  **SECONDARY OUTCOMES**: Total SCARED scores (CHILD rated) were significantly lower at post-treatment (Mdn = 19) than pre-treatment (Mdn = 24) z= -3.44, p=0.001, r=0.85 (large effect size). Scores were significantly lower for 4 - 5 SCARED subdomains (effect size): panic disorder/somatic symptoms (0.26), generalised anxiety (0.15), separation anxiety (0.28) and school avoidance (0.08), but not for social anxiety (0.08, P = ns) . 44% of students who met clinical criteria at pre-test (based on total SCARED score), no longer met criteria at post-treatment assessment - this was a statistically significant reduction x2 (1, N=35) = 5.4, p=0.02).  **PARENT rated SCARED scores -** there was a significant reduction in total score from pre (Mdn= 20) to post treatment (Mdn = 17) z= -2.20, p=.0.03, r=0.44 (medium effect size). Three out of the 5 domains were significantly lower at post-treatment: generalised anxiety (0.15), separation anxiety (0.13) and school avoidance (0.06) (small effect sizes). Panic/somatic and social anxiety did not significantly reduce. //Total SCARED (parent) scores, showed that there was no significant difference in terms of the proportion of children meeting clinical cut off for anxiety pre to post intervention x2 (1, N=35), =0.67, p=0.41.  **Teacher ratings on the DBC-T** showed that total scores were not significantly lower at post-treatment (M=20.4) compared with pre-treatment (M=20.9). z=-1.30, p=0.20, r= -0.15. Subdomain scores did not significantly change pre-post treatment, nor the proportion of students meeting clinical cut offs on the Total DBC-T scores (presence of emotional/behavioural problems). |
| Yeo, 2016 | **PRIMARY OUTCOMES**: NONE -  **SECONDARY OUTCOMES**: **INTERVENTION IMPACT ON TEST ANXIETY:** *2 months post intervention* - CBT group reported lower anxiety levels than the control group. Mean change in anxiety scores (pre intervention to follow up) was significantly greater for the intervention group (M=0.26, SD = 0.60) compared with control (M = -0.01, SD = 0.41), t (113) = -2.74, p = .007, two tailed)."The magnitude of the differences in the mean change in test anxiety scores (mean difference -0.27, 95 % CI -0.46 to -0.07) was medium (d = 0.52)."  There was a significant time by group interaction indicating that test anxiety scores changed over time - there was no change over time within the control group but the CBT group scored significantly lower at follow up compared to baseline and post treatment**.** There was no significant change in test anxiety in the CBT group between baseline and post-treatment however.  **TEST ANXIETY AS A MODERATO**R: - when participants were grouped into high baseline test anxiety (above the median) and low baseline test anxiety (below the median), there was a significant difference post-treatment and at follow up for the high severity group only, "as evidenced by the large interaction effect [Wilks’ Lambda = 0.72, F(2, 51) = 10.14, p \ .0001, partial eta squared = 0.29] and large group effect [F(1, 52) = 20.13, p \ .0001, partial eta squared = 0.28.]."  **EFFECT OF INTERVENTION ACCORDING TO ACHIEVEMENT 'LEVEL' (high, average, low)** " For the control group, a main effect was registered for achievement groups only. There were large differences in test anxiety scores for high, average, and low achievers who received no intervention. As predicted, average achieving students reported increasing test anxiety whereas their high and low achieving peers were fairly consistent in their anxiety levels. For the CBT group, only time effect was significant, i.e., test anxiety was reduced over time for all students. The impact of the intervention was most evident between post-treatment and two-month follow-up as indicated by a substantial main effect for time, Wilks’ Lambda = 0.88, F(1, 55) = 7.37, p = .009, partial eta squared = 0.12."  **TESTING WHETHER COGNITIVE-BEHAVIOURAL SKILLS CONTRIBUTE TO OUTCOMES** The authors predicted that interventions with behavioural elements would lower test anxiety. Cognitive-behavioral skills (CBSC) scores reported at follow-up were added as a covariate in the mixed-design ANOVA to examine the effects of treatment. We had earlier found significant reduction in test anxiety scores between post-treatment and follow-up. When the CBSC scores were included as a covariate, we were ‘‘removing’’ the CBT skills that were taught and therefore, we expected there would no longer be significant differences in test anxiety scores between post-treatment and follow-up. After controlling for CBT skills, there was no significant interaction effect, Wilks’ Lambda = 0.97, F(2, 54) = 0.79, p = .46, partial eta squared = 0.03. As anticipated, there was also no main effect for time, Wilks’ Lambda = 0.99, F(1, 54) = 0.72, p = .40, partial eta squared = 0.01. The difference in test anxiety scores across the two time periods was no longer significant. The main effect for achievement groups comparing the three academic achievement groups was also not significant, F(2, 54) = 0.94, p = .40, partial eta squared = 0.03. Thus, when we filtered out cognitive-behavioral skills practice, the change in anxiety between post-treatment and follow-up was no longer significant." **DETERMINING WHICH COGNITIVE BEHAVIOURAL SKILLS WERE ACTIVE IN TREATMENT:** Scores on each of the seven skills in the CBSC were used as a covariate to control for the practice of that specific skill, to see whether this produced non-significant outcomes at follow up. All but one of the seven skills—using calming self-talk— did not contribute to the positive treatment outcomes. |
| Phuphaibul 2003 | **Primary outcomes - Coping (YA-COPE):** The mean coping score for experimental group 1 increased from 110.87 to 114.92 at one month follow up (not statistically tested) whereas the mean coping score for experimental group II slightly decreased from pre (112.96) to 1 month follow up (111.73) (not statistically tested). The mean coping score for the control group also slightly decreased from 12.43 to 109.90 at 1 month follow up (not statistically tested).// As predicted, after controlling for pre-test coping score, there was a significant difference between coping scores in experimental groups 1 and 2 at one month follow up (F=5.135, p<0.05), with the intensive teacher training group achieving better outcomes.  **Secondary outcomes - mental health -** The mean mental health scores in experimental group 1 decreased (lower score = fewer mental health problems or stress) slightly from 0.99 to 0.97 at one-month follow up (not statistically tested). Mean mental health scores in experimental group 2 decreased slightly from 1.00 to 0.94 at one month follow up. There was almost no change in the control group. // Contrary to predictions, after controlling for pre-test score differences, there was no significant difference between mental health scores of the two experimental groups (F = 0.87, P = 0.352). |
| Phuphaibul 2005 | **Primary outcomes - coping (YA-COPE) -** As predicted, after controlling for pre-test scores, a statistically significant difference at post-test was found between the control and experimental group 1 , with the experimental group showing better coping skills than the control (F=9.425, p<0.01).  As predicted, after controlling for pre-test scores, a statistically significant difference at one month was found between the control group and experimental group 2, with the experimental group showing better coping skills than the control group (F = 22.446, p <0.001).  **Secondary outcomes - mental health:** As predicted, after controlling for the pre-test score, a statistically significant difference at 1 month was found between scores in the control group and experimental group 1 (F= 6.034, p < 0.05).  As predicted, after controlling for the pre-test score, a statistically significant difference at 1 month was found between scores in the control group and experimental group 2, with better mental health outcomes in the experimental group compared with the control. |
| Jegannathan 2014 | **Primary outcomes- Life Skills Dimension scores : ALL YP:** When broken down by gender, girls showed mild-moderate improvement (based on effect size) improvement post intervention in terms of their interpersonal communication/human relationship dimension (0.45), physical fitness/health maintenance (0.20) and total life skills (0.24). Boys only improved in the interpersonal communication/human relationship dimension (0.36). *Statistics for dimensions that did not show improvement are not reported.*  BOYS WITH HIGH RISK showed mild-moderate improvement in interpersonal communication/human relationship (0.48), identity development/purpose in life (0.26) and total life skills dimension scores (0.22), however decision making (0.18) and health maintenance (-0.16) scores showed little or no effect.  GIRLS WITH HIGH RISK showed little or no improvement across any dimension (scores all <0.2): human relationship (0.19), decision making (-0.09), health maintenance (0.15), purpose in life (-0.07), total score (0.09).  **Secondary outcomes** - **Mental health profile scores** - Youth Self Report (YSR). When ALL youth were analysed, effect sizes from the intervention on YSR sub-scale scores **(anxious/depressed,** withdrawn, somatic complaints, social problems, attention problems, rule-breaking behaviour, aggressive behavior, internalising and externalising) were 'small'/'no effect' (all <0.2).  When pre-post difference YSSR scores were compared, effect sizes amongst HIGH RISK BOYS were > 0.2 (classed as 'small effect') across the following YSR sub-scales: withdrawn (0.40), attention problems (0.46), rule breaking behaviour (0.36), aggressive behaviour (0.48) and externalising syndrome (0.64). They were however < 0.2 across the remaining scales, i.e. **anxious/depressed (0.13)**, somatic complaints (0.09), social problems (0.09) internalising (0.14). In 'high risk' GIRLS, effect sizes were lower than 0.2 across every YSR domain: **anxious/depressed (0.00)**, withdrawn (-0.23), somatic complaints (0.07), social problems (-0.02), attention problems (-0.13), rule breaking behaviour (-0.38), aggressive behaviour (0.06), internalising (-0.04), externalising (-0.13). |
| Monkong 2009 | **Primary outcomes:** Following intervention, participants receiving the intervention "showed significant improvement of knowledge, attitude and practice (P<0.05)". **Secondary outcomes:** Following intervention, there was a statistically significant reduction in levels of stress (P<0.05). |

**Table S5: Quality**

|  | **Quantitative experimental** | | |  | **Mixed methods** | | |  | **Qualitative** | | | | | |
| --- | --- | --- | --- | --- | --- | --- | --- | --- | --- | --- | --- | --- | --- | --- |
| **STUDY ID** | **1** | **2** | **3** |  | **1** | **2** | **3** |  | **1** | **2** | **3** | **4** | **5** | **6** |
| Drmic 2017 | 0 | 0 | 1 |  | 0 | 1 | 1 |  | 1 | 1 | 1 | 1 | 0 | 0 |
| Yeo, 2016 | 0 | 0 | 1 |  | N/A | N/A | N/A |  | N/A | N/A | N/A | N/A | N/A | N/A |
| Phuphaibul 2003 | unclear | 0 | unclear |  | N/A | N/A | N/A |  | N/A | N/A | N/A | N/A | N/A | N/A |
| Phuphaibul 2005 | unclear | 0 | unclear |  | N/A | N/A | N/A |  | N/A | N/A | N/A | N/A | N/A | N/A |
| Jegannathan 2014 | unclear | 0 | 1 |  | N/A | N/A | N/A |  | N/A | N/A | N/A | N/A | N/A | N/A |
| Monkong 2009 | 0 | 0 | unclear |  | N/A | N/A | N/A |  | N/A | N/A | N/A | N/A | N/A | N/A |
